# A Pectic Polysaccharide from *Codonopsis pilosula* Alleviates Inflammatory Response and Oxidative Stress of Aging Mice via Modulating Intestinal Microbiota-Related Gut–Liver Axis

**DOI:** 10.3390/antiox12091781

**Published:** 2023-09-19

**Authors:** Yuanfeng Zou, Hong Yan, Cenyu Li, Fang Wen, Xiaoping Jize, Chaowen Zhang, Siqi Liu, Yuzhe Zhao, Yuping Fu, Lixia Li, Fan Liu, Ji Chen, Rui Li, Xingfu Chen, Mengliang Tian

**Affiliations:** 1 Natural Medicine Research Center, College of Veterinary Medicine, Sichuan Agricultural University, Chengdu 611130, Chinalixiali@sicau.edu.cn (L.L.); 2 College of Agronomy, Sichuan Agricultural University, Chengdu 611130, China; liufantl2006@163.com (F.L.);

**Keywords:** polysaccharide, aging, inflammation, oxidative stress, intestinal microbiota, gut–liver axis

## Abstract

Aging is a biological process that leads to the progressive deterioration and loss of physiological functions in the human body and results in an increase in morbidity and mortality, and aging-related disease is a major global problem that poses a serious threat to public health. Polysaccharides have been shown to delay aging by reducing oxidative damage, suppressing inflammatory responses, and modulating intestinal microbiota. Our previous studies have shown that polysaccharide CPP-1 extracted from the root of *Codonopsis pilosula* possesses noticeable anti-oxidant activity in vitro. Thus, in our study, we tested the anti-aging effect of CPP-1 in naturally aging mice (in vivo). Eighteen C57/BL mice (48-week-old, male) were divided into a control group, high-dose CPP-1 group (20 mg/mL), and low-dose CPP-1 group (10 mg/mL). We discovered that CPP-1 can exert a reparative effect on aging stress in the intestine and liver, including alleviating inflammation and oxidative damage. We revealed that CPP-1 supplementation improved the intestinal microbiota composition and repaired the intestinal barrier in the gut. Furthermore, CPP-1 was proved to modulate lipid metabolism and repair hepatocyte injury in the liver by influencing the enterohepatic axis associated with the intestinal microbiota. Therefore, we concluded that CPP-1 prevents and alleviates oxidative stress and inflammatory responses in the intestine and liver of aging mice by modulating the intestinal microbiota-related gut–liver axis to delay aging.

## 1. Introduction

Aging is a process of the gradual deterioration of the body’s tissues and organs. It is accompanied by the decline and disappearance of all organ functions, eventually leading to cellular and individual death [1]. Along with the aging process, related diseases such as cardiovascular diseases, cancer, and Alzheimer’s disease are increasing globally and therefore delaying aging has become an inevitable trend [2]. Currently, many anti-aging theories have been proposed regarding various modes of aging effects, the most famous of which is the free-radical theory [3]. This theory illustrates that oxidative stress is the primary inducer of aging, which leads to an imbalance in the production and consumption of reactive oxygen species (ROS). A high level of ROS accumulation and the declining levels of anti-oxidant enzymes that can scavenge ROS could cause oxidative damage and chronic inflammation in cells and organs, represented as typical aging features [4,5,6].

One of the main aging characteristics of the body is the progressive loss of intestinal function, as evidenced by the damage on the intestinal mucosa, reduced immune function, and disturbances of the intestinal flora [6,7]. Intestinal dysfunction also leads to the entry of certain microorganisms and pathogens (LPS, for instance) into the systemic circulation that reach the liver, which causes liver detoxification or metabolic disorders and the disruption of liver function, further affecting the intestine through the gut–liver axis [8]. Increasing studies have been performed on treating aging-related diseases with intestinal microbes, including dietary probiotics and other emerging therapeutic applications such as fecal microbiota transplantation [9]. As a result, it was hypothesized that hepatic and intestinal diseases could be alleviated via the gut–liver axis and intestinal bacteria. And the further screening of new drugs that could delay hepatic and intestinal aging through the enterohepatic axis is essential, which could be highly associated with the modulation of the intestinal flora.

Several medications have been found to have effective anti-aging properties. However, most of them still have some drawbacks on safety and show side effects after long-term use. For instance, metformin and rapamycin have been demonstrated to possess strong anti-aging effects in various model organisms via different ways [10,11]. Long-term use of metformin may lead to the lack of vitamin B12 and the accumulation of lactic acid [12], while rapamycin may cause thrombocytopenia, nephrotoxicity, and other side effects [13]. Thus, it is critical to investigate herbal remedies that are naturally effective, free of side effects, and safe for elders to use in the long-term. *Codonopsis pilosula,* also known as Dangshen, is a traditional Chinese herbal medicine that is widely cultivated in Asian countries for both food production and medicinal treatment [14]. The root of *C. pilosula* has been used as a traditional Chinese medicine for thousands of years. Modern pharmacological studies have shown that *C. pilosula* possesses numerous medical effects, including improving memory impairment [15], alleviating oxidative stress [16], enhancing immunity [17], and protecting the digestive system [18]. *C. pilosula* polysaccharides (CPs), as the main water-soluble components of *C. pilosula*, exhibit a variety of pharmacological effects such as anti-tumor [19], immunomodulatory [20], anti-inflammatory [21], anti-oxidative, and anti-aging effects [22]. Meanwhile, CPs have been shown to be promising drugs for the treatment of various diseases and functional components in food preparations. It has also been proved that polysaccharides are primarily digested in the intestine and degraded by gut microbiota; thus, the intestine is the major target organ of CPs [23]. Moreover, CPs have fewer side effects and lower cytotoxicity and are thus allowed for long-term use [24]. These advantages give a clue that the long-term use of CPs may protect and prevent aging of the liver and intestine by modulating the enterohepatic axis associated with the intestinal flora. In our previous study, a polysaccharide fraction isolated from the roots of *C. pilosula* (CPP-1) was proved that could alleviate oxidative stress in intestinal porcine enterocyte cells (IPEC-J2 cells) by modulating the Nrf2 pathway [22]. Meanwhile, CPs have excellent anti-inflammatory effects in vivo [21].

Therefore, we selected the anti-inflammatory and anti-oxidative capacity as the major indicators for evaluating the anti-aging ability of CPP-1 in the intestine and liver. In addition, through the microbiota-related gut–liver axis, we assessed the composition of intestinal microbes and the shifts in the liver functions to evaluate the effect of CPP-1 in the circulation. Our study provided a theoretical basis for researching natural anti-aging drugs for long-term use.

## 2. Methods

### 2.1. Plant Source

*C. pilosula* roots were collected from Yongxin County (Gansu Province, China) and identified by Dr. Li-Xia Li, College of Veterinary Medicine, Sichuan Agricultural University. The characteristics of dry roots were consistent with the description in the Pharmacopoeia of the People’s Republic of China [14]. The polysaccharide fraction from *C. pilosula* roots was extracted and purified according to our previous study [22], named CPP-1, in which the primary structural characterization of CPP-1 was determined [22].

### 2.2. Animal Care and Experimental Design

Animal experiments were conducted under the supervision of the Ethics Committee for Animal Experiment at Sichuan Agriculture University (Confirmation number: DYXY141642008). Eighteen C57/BL mice (48-week-old, male), weighing approximately 30 g, were purchased from Charles River Laboratories and maintained for 1 week to adapt to the new environment (22 ± 2 °C).

Mice were randomly divided into three groups: (a) normal control group, (NC, gavage saline, *n* = 6), (b) low-dosage CPP-1 group (gavage 10 mg/kg CPP-1 in saline, *n* = 6) group, and (c) high-dosage CPP-1 group (gavage 20 mg/kg CPP-1 in saline, *n* = 6). The saline and CPP-1 solutions were intragastrically supplied for 14 days and the body weights were measured every 3 days. Furthermore, the body appearance, appetite, mental condition, and behavior of mice were observed and recorded every day. After 14 days of administration with CPP-1 or saline, mice were euthanized with carbon dioxide followed by cervical dislocation, and blood, tissues (intestine and liver), cecal contents were collected for further determination.

### 2.3. RNA Extraction and Quantitative Real-Time Polymerase Chain Reaction (qPCR)

The total RNAs from intestine tissues (duodenum, jejunum, ileum) and liver were extracted with TRIzol reagent by following the instructions (Biomed, RA-101-02, Beijing, China). The isolated total RNAs were reversely transcribed into cDNAs using the M-MLV4 First-Strand cDNA Synthesis Kit (Takara Biotechnology, Co., Ltd., Dalian, China) after measuring the RNA concentration with a microspectrophotometer (Thermo Scientific, NanoDrop^TM^ One/OneC, Waltham, MA, USA). Real-time PCR was performed using the SYBR premix Ex Taq. II Kit (Takara Biotechnology, Co., Ltd., Dalian, China) and the results of mRNA expression were calculated via 2^−ΔΔCt^ method and normalized to β-actin expression. The primers for superoxide dismutase (SOD), glutathione peroxidase (GPX), catalase (CAT), nuclear factor erythroid-2-related factor 2(Nrf2), Interleukin-1β (IL-1β), tumor necrosis factor-α (TNF-α), Interleukin-6 (IL-6), toll-like receptor4 (TLR4), zonula occludens protein 1 (ZO-1), occludin, Mucin2 (MUC2), and β-actin are listed in Table 1.

### 2.4. Hematoxylin and Eosin Staining of Liver and Jejunum (H&E Staining)

The liver and jejunum were embedded in paraffin and sliced (5 μm), and then the sections were stained with hematoxylin and eosin (Hematoxylin-Eosin Staining Kit, Beijing Solarbio Science and Technology Co., Ltd., Beijing China). Sections were photographed with Nikon eclipse 80i microscope (Nikon Instruments, Melville, NY, USA) at 200× magnification.

### 2.5. The Determination of Anti-Oxidative Effect of CPP-1 during Aging Process in Mice

To study the anti-oxidative effect of CPP-1 on aging mice, the levels of SOD, GPX, CAT, total anti-oxidant capacity (T-AOC), and malondialdehyde (MDA) were measured using commercially available biochemical kits according to the manufacturer’s instructions (Nanjing Jiancheng Biotechnology Institute, Nanjing, China). The level of reactive oxygen species (ROS) was evaluated by using ELISA kits (Quanzhou Ruixin Biotechnology Co., Ltd., Quanzhou, China). The detailed description is provided in Table S1.

### 2.6. The Determination of Anti-Inflammatory Effect of CPP-1 during Aging Process in Mice

To investigate the efficacy of CPP-1 on alleviating chronic inflammation in aging mice, the levels of IL-6, IL-1β, and TNF-α in the liver, jejunum, and serum were determined by ELISA kits according to the instructions (Quanzhou Ruixin Biotechnology Co., Ltd., China).

### 2.7. Determination of LPS in Serum, Liver, and sIgA in the Jejunum

The level of serum lipopolysaccharide (LPS) was determined using ELISA kits according to the manufacturer’s instructions (Quanzhou Ruixin Biotechnology Co., Ltd., China). Jejunum and liver samples were ground with liquid nitrogen and suspended in 10-fold volumes of PBS. The supernatants were taken to determine the level of secretory immunoglobulin (sIgA) and LPS using ELISA kits according to the manufacturer’s instructions (Quanzhou Ruixin Biotechnology Co., Ltd., China).

### 2.8. Serum Biochemical Parameter Analysis

Alanine aminotransferase (ALT); aspartate aminotransferase (AST); high-density lipoprotein cholesterol (HDL-C); low-density lipoprotein cholesterol (LDL-C); total cholesterol (TC); and triglyceride (TG) levels in the serum as markers of liver damage were evaluated via a dry chemistry analyzer (Fujifilm, Tokyo, Japan).

### 2.9. Gut Microbiota Analysis

Fresh feces (150 mg) of mice were collected and analyzed by Novogene Science and Technology Co., Ltd. (Beijing, China). The DNA was extracted using cetyltrimethylammonium bromide (CTAB)-based method and diluted to 1 ng/μL by sterile water. The 16S rRNA genes of the V4 regions were amplified using specific primer (F: GTGCCAGCMGCCGCGGTAA; R: GGACTACHVGGGTWTCTAAT) with barcode, and the PCR products were purified using Qiagen Gel Extraction Lit (Qiagen, Hilden, Germany). Sequences with ≥97% similarity were assigned to the same operational taxonomic units (OTUs). Alpha and beta diversity analysis were performed to evaluate intestinal microbiota diversity and differences. Cluster analysis was performed via principal components analysis (PCA) and non-metric multi-dimensional scaling (NMDS) by R 4.2.0 software. Different and enriched bacterial species among groups were presented by MetaStat and LDA effect size (LEfSe) with statistical analysis of *t*-test.

### 2.10. Statistical Analysis

In addition to the statistical analysis used in metabolomic and microbiota experiments, the rest of the data were expressed as mean ± standard deviation (S.D) and analyzed using one-way analysis for variance and LSD test (IBM SPSS Statistics version 24, IBM Corp., Armonk, New York, NY, USA). *, *p* < 0.05 versus control group, ** *p* < 0.01 versus control group, *** *p* < 0.001 versus control group, and # *p* < 0.05 versus 10 mg/kg CPP-1 group, ## *p* < 0.01 versus 10 mg/kg CPP-1 group, ### *p* < 0.001 versus 10 mg/kg CPP-1 group. Spearman analysis was performed by R 4.2.0. corrplot package (*p* < 0.05).

## 3. Results

### 3.1. CPP-1 Protected Naturally Aging Mice from Oxidative Stress and Inflammation

**Figure 1:**
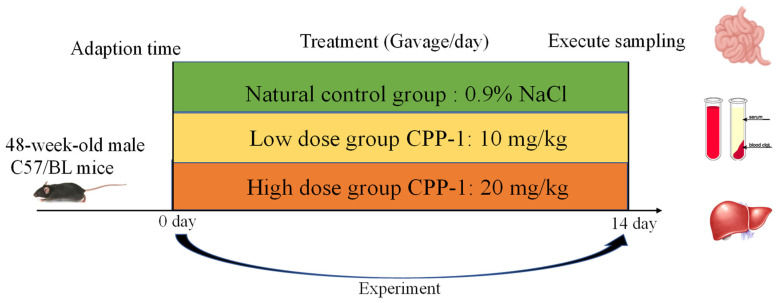
Schematic overview of experimental design.

**Figure 2:**
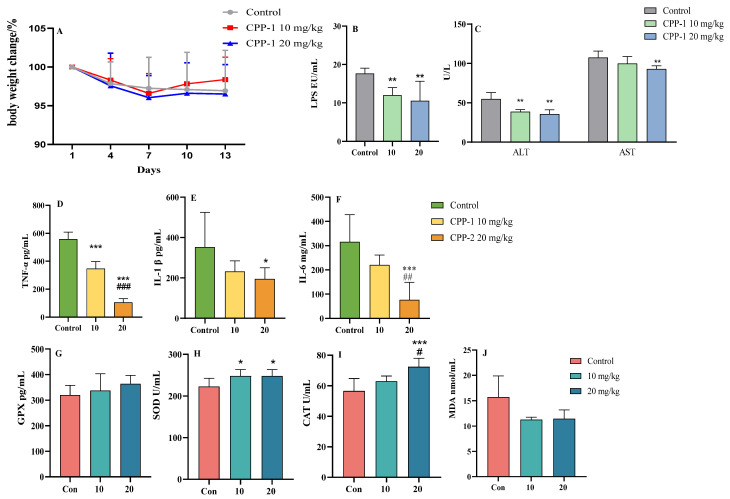
Effects of CPP-1 on body weight and the levels of LPS, ALT, AST, pro-inflammatory factors (IL-6, IL-1β, TNF-α), anti-oxidative enzymes (SOD, GPX, CAT), and MDA in serum. (**A**) Body weight changes in mice (relative to initial body weight); (**B**) the serum level of LPS; (**C**) the serum levels of AST and AST; the serum levels of pro-inflammatory factors (**D**) TNF-α, (**E**) IL-6, (**F**) IL-1β; the activity of anti-oxidative enzymes, (**G**) GPX, (**H**) SOD, (**I**) CAT, and the level of (**J**) MDA. Error bars indicate SEM (*n* = 6). Differences are statistically significant at * *p* < 0.05, ** *p* < 0.01, *** *p* < 0.001 compared with control group, and # *p* < 0.05, ## *p* < 0.01, ### *p* < 0.001 compared with 10 mg/kg CPP-1 group.

A schematic figure of the animal experimental design is shown in Figure 1. Different dosages of CPP-1 (10 mg/kg and 20 mg/kg) were supplied intragastrically to naturally aging mice. After 14 days of feeding, there was no significant difference in the body weights among the natural control mice and different doses of the CPP-1-supplied mice (Figure 2A). As shown in Figure 2B,C, we determined the serum LPS levels and ALT and AST activities to examine whether CPP-1 has a reparative effect on the intestinal and liver injury caused by natural aging. It suggested that CPP-1 significantly reduced serum LPS levels and enhanced ALT and AST activities, indicating CPP-1 protected naturally aging mice from liver and intestinal damage induced by aging. In addition, CPP-1 significantly reduced the inflammatory factors (TNF-α, IL-1β, and IL-6, Figure 2D–F) and MDA (Figure 2J) in the serum of the natural control group mice, while CPP-1 also increased the activities of impaired anti-oxidant enzymes (GPX, SOD, and CAT, Figure 2G–I) in a dose-dependent manner, with superior effects in the high-dose group. It suggested that different doses of CPP-1 exhibited great anti-oxidative and anti-inflammatory activities and may have had ameliorative effects on liver and intestinal injuries. Thus, we will study the protective effects of CPP-1 on oxidative stress and inflammation in the intestine and liver of naturally aging mice.

### 3.2. CPP-1 Attenuated Oxidative Stress and Inflammation in the Gut of Naturally Aging Mice

**Figure 3:**
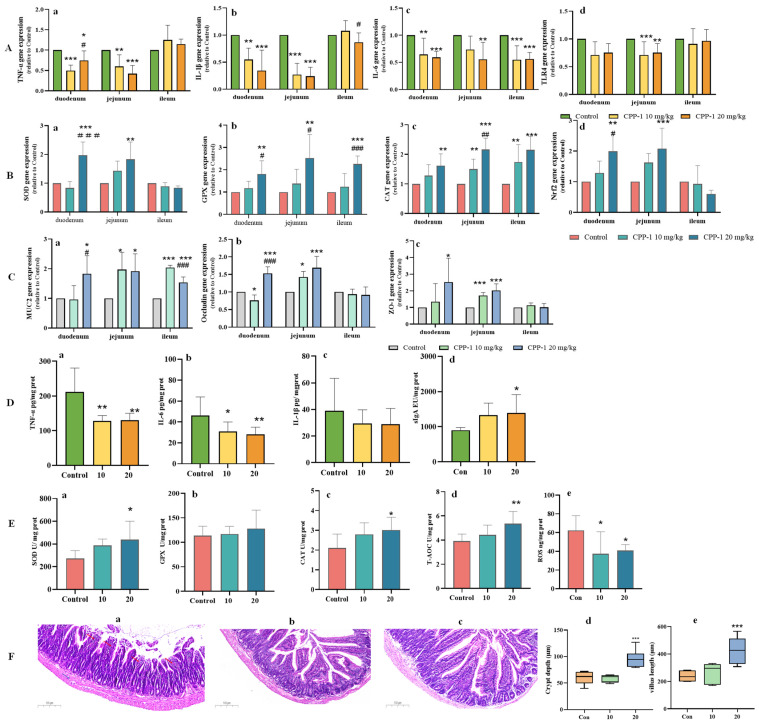
Effects of CPP-1 on anti-inflammatory, anti-oxidant, and intestinal barrier protection capacity of intestine in naturally aging mice. (**A**) The gene expressions of inflammatory factors in intestine (duodenum, jejunum, and ileum), (**a**) TNF-α, (**b**) IL-1β, (**c**) IL-6, (**d**) TLR4. (**B**) The gene expressions of anti-oxidative enzymes (duodenum, jejunum, and ileum), (**a**) SOD, (**b**) GPX, (**c**) CAT, (**d**) Nrf2. (**C**) The gene expressions of intestinal barrier, (**a**) MUC2, (**b**) occludin, (**c**) ZO-1. (**D**) The protein levels of inflammatory factors and sIgA in jejunum, (**a**) TNF-α, (**b**) IL-6, (**c**) IL-1β, (**d**) sIgA. (**E**) The protein levels of anti-oxidative enzymes and ROS in jejunum, (**a**) SOD, (**b**) GPX, (**c**) CAT, (**d**) T-AOC, (**e**) ROS. (**F**) The histopathological staining of jejunum of naturally aging mice, (**a**) control, (**b**) CPP-1 10 mg/kg, (**c**) 20 mg/kg. Red arrow: inflammatory cell infiltration; blue arrow: intestinal mucosa thickening. (**d**,**e**) Quantification demonstration of villus and crypt in jejunum. Error bars indicate SEM (*n* = 6). Differences are statistically significant at * *p* < 0.05, ** *p* < 0.01, *** *p* < 0.001 compared with natural control group, and # *p* < 0.05, ## *p* < 0.01, ### *p* < 0.001 compared with 10 mg/kg CPP-1 group.

CPP-1 could significantly down-regulate the levels of serum inflammatory factors and up-regulate the activity of serum anti-oxidative enzymes. Polysaccharides, however, as macromolecules, are primarily utilized in the intestine [25]. And the intestinal permeability index LPS that was detected in the serum was significantly inhibited by CPP-1, suggesting that CPP-1 has a protective effect on the intestine of aging mice. The intestine is the major place for the body to absorb nutrition, making it an important target organ for delaying aging and prolonging life [26]. Intestinal aging will lead to negative changes on the intestinal physiological structure and function, such as inflammation and oxidative stress and mucosal barrier dysfunction, which will cause aging degeneration on other organs [27]. Therefore, we studied the intestinal protective effects of CPP-1 in naturally aging mice based on the anti-inflammatory, anti-oxidant, and intestinal barrier protective effects. The qPCR results suggested that CPP-1 decreased the gene expressions of IL-6, IL-1β, TNF-α, and TLR4 (Figure 3A) and increased the gene expressions of SOD, GPX, CAT, and Nrf2 in the intestine compared with the natural control group mice (Figure 3B). In addition, CPP-1 was shown to restore the intestinal barrier, including enhancing the gene expressions of MUC2, occludin, and ZO-1 (Figure 3C). However, the anti-inflammatory, anti-oxidative, and barrier-restoring properties varied with different intestine segments. Regarding the anti-inflammatory activity, the levels of IL-1β, TNF-α, and TLR4 in the ileum had no significant difference among the control and CPP-1 treatment groups (*p* > 0.05), as shown in Figure 3A, whereas in the duodenum and jejunum, the levels of TNF-α, IL-1β, IL-6, and TLR4 presented in a dose-dependent decrease, and the effect of CPP-1 was the most significant in the jejunum (*p* < 0.05). The difference was also shown in the anti-oxidative aspect (Figure 3B), as CPP-1 only significantly elevated the gene expressions of CAT and GPX compared with the control group in the ileum. In the duodenum and jejunum, the high-dose CPP-1 significantly enhanced the gene expressions of all anti-oxidant enzymes SOD, CAT, and GPX, and the signaling factor Nrf2, with the latter being more prominent. These results suggested that the anti-oxidant effect exerted by CPP-1 was mainly in the anterior part of the small intestine. We also determined the gene expression levels of several common gut barrier factors (MUC2, occludin, and ZO-1) to evaluate the intestinal barrier function. As shown in Figure 3C, the gene expressions of MUC2 (a) in the duodenum and jejunum were significantly up-regulated by CPP-1. In terms of the expression of occludin (b) and ZO-1 (c) in the ileum, there was no difference between the CPP-1 groups and control group (*p* > 0.05); however, a significant enhancement in its expression in the jejunum and duodenum by CPP-1 was observed (Figure 3C). It suggested that CPP-1 could repair the intestinal barrier function in a dose-dependent manner, and it behaved the best in the jejunum.

As a result, by measuring the function of CPP-1 on the repair of the inflammatory responses, oxidative stress, and intestinal barrier in different intestinal segments of aging mice at the gene level, it seems that CPP-1 had the most significant effect on the jejunum. We further evaluated the anti-inflammatory and anti-oxidative activities of CPP-1 at the protein level. As shown in Figure 3D, CPP-1 was shown to attenuate inflammatory stress in the jejunum of aging mice by reducing inflammatory factors TNF-α (a), IL-6 (b), and IL-1β (c). The content of sIgA (d), an immune marker, increased in the jejunum with CPP-1 supplementation compared with the natural control group, suggesting that CPP-1 repaired the intestinal immune barrier in naturally aging mice. From Figure 3E, CPP-1 treatment enhanced the activity of SOD (a), GPX (b, *p* > 0.05), CAT (c), and T-AOC (d), and reduced the ROS (e) in the jejunum of aging mice. Meanwhile, we evaluated the repairing effect of CPP-1 on the intestine by observing intestinal pathological changes via H&E staining. As shown in Figure 3F(a–c), the ageing mice exhibited increased inflammatory cell infiltrating (red arrow) and thickened mucosa (blue arrow) with reduced villus and crypt (Figure 3F(d,e)). In contrast, the gavage of CPP-1 (particularly at 20 mg/kg) contributed to the improvement in the various lesions mentioned above, including thinning of the intestinal mucosa, reduction of inflammatory cells, and restoration of the shape and number of crypt and villi.

### 3.3. CPP-1 Modulated the Diversity and Composition of Intestinal Microbiota of Aging Mice

**Figure 4:**
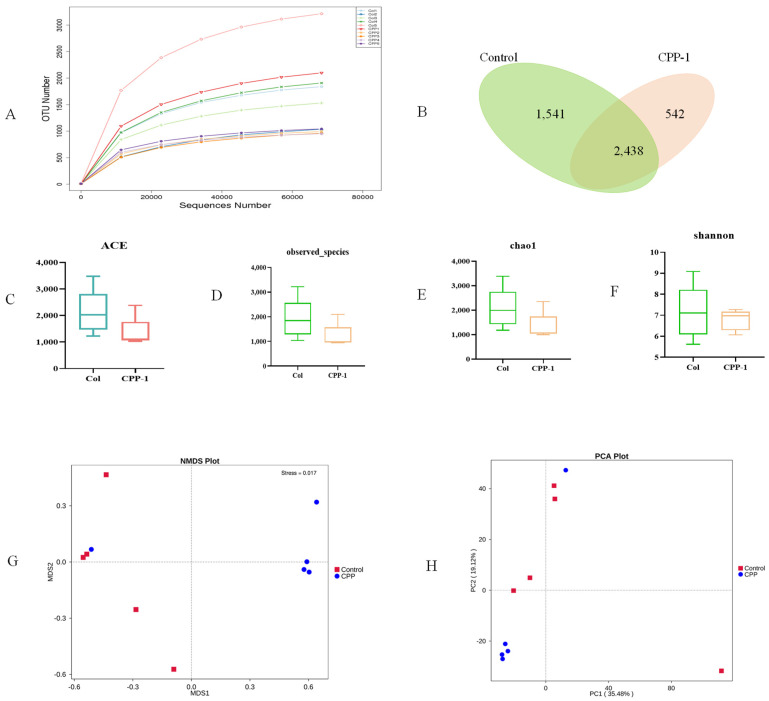
CPP-1 polysaccharide modulated the composition and structure of gut microbiota. CPP stands for high-dose CPP-1 group. (**A**) OTU-rarefaction curves of gut microbiota in different groups; (**B**) Venn diagram showing the unique and shared OTUs from groups; bacterial community richness measured by (**C**) ACE, (**D**) observed_species, (**E**) chao1, (**F**) Shannon indexes in different groups; (**G**) NMDS diagram based on Bray–Curtis similarities of bacterial communities; (**H**) PCA diagram based on Euclidean distances.

During aging, oxidative and inflammatory stress affect the survival environment of the intestinal microbiota, disrupting the composition and function of bacteria and leading to an inequilibrium of the gut microbe ecosystem [28]. Polysaccharides, as large molecules, are generally not directly digested and absorbed by the body but are degraded by the intestinal flora to produce a series of beneficial changes that affect the body [29]. Therefore, we analyzed the fecal microbiota composition of the natural control group mice and high-dose CPP-1 group mice (better effect as demonstrated by the above results) to reveal the changes and patterns of gut microbiota during CPP-1-delayed aging. As shown in Figure 4A, the OTU rarefaction curve reached a stable level, indicating that the libraries could obtain the primary information on the bacterial diversity of all samples. The number of OTUs in the natural control and high-dose CPP-1 groups were 3979 and 2980, respectively, and possessed 2438 identical OTUs between these two groups, indicating that CPP-1 reduced the total number of OTUs in the intestinal flora of naturally aging mice (Figure 4B). According to the alpha analysis, including ACE, observed species, chao1, and Shannon index (Figure 4C–F), age-related stress increased the richness of gut microbial species, while CPP-1 decreased the diversity of the gut microbes. In addition, the NMDS and PCA analysis revealed a clear segregated clustering of gut microbiota among naturally aged and CPP-1-treated mice, suggesting that the structure and composition of the gut microbes of aging mice were altered by CPP-1 (Figure 4G,H).

**Figure 5:**
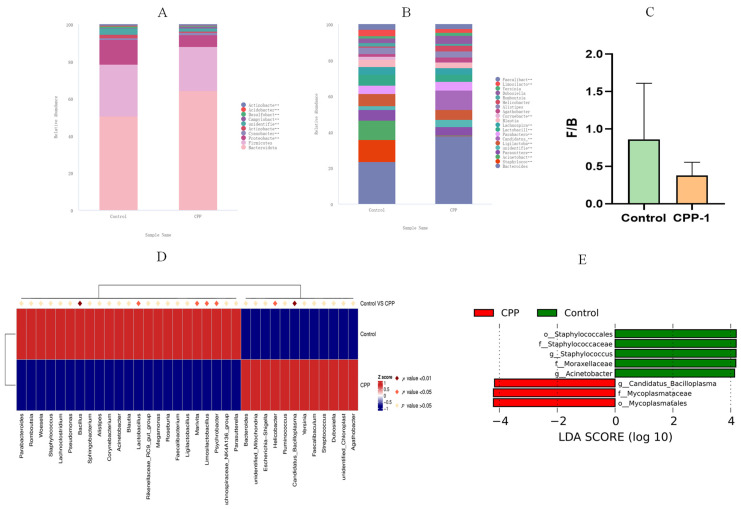
Comparative analysis of the effects of CPP-1 supplementation on the gut microbiota. CPP stands for high-dose CPP-1 group. The distribution of intestinal microbiota at the phylum level ((**A**), top 10 bacteria) and genus level ((**B**), top 20 bacteria); (**C**) quantification shows the abundance ratio of *Firmicutes*/*Bacteriodota*; (**D**) the MetaStat analysis shows the microbes with significant differences in abundance in different groups; (**E**) the histogram of the distribution of LDA score shows the microbes with significant differences in abundance in different groups.

We further studied the taxonomic distribution of abundant bacteria at the phylum and genus levels. Microbial community analysis at the phylum level and genus level revealed a different bacterial community structure between the groups. At the phylum level (Figure 5A), the intestinal microbes of the natural control group mice primarily composed of *Bacteroidota* (48.33%), *Firmicutes* (26.87%), and *Proteobacteria* (12.66%), accounting for about 90% of all intestinal flora in aging mice. Studies have shown that age-related stress is associated with significant increases in the ratio of *Firmicutes*/*Bacteriodota* (F/B, Figure 5C) and *Proteobacteria* [30]. However, the intestinal microbiota structure of the CPP-1-treated mice was altered, with a decrease in the F/B value and *Proteobacteria*, which was consistent with the results of a published literature [31]. As for the bacteria at the genus level (Figure 5B), the intestinal flora of the natural control group mice primarily composed (over 1%) of *Bacteroides* (7.66%), *Staphylococcus* (3.93%), *Acinetobacter* (3.50%), *Parasutterella* (1.90%), *Ligilactobacillus* (2.20%), *Parabacteroides* (1.50%), *Lactobacillus* (2.00%), *Lachnospiraceae_NK4A136_group* (1.41%), *Blautia* (1.27%), and *Alistipes* (1.20%). The CPP-1 remedy affected the abundance and composition of the intestinal flora, including an increase (over 0.1%) in *Bacteroides* and a decrease (over 0.1%) in *Staphylococcus*, *Acinetobacter*, *Parasutterella*, *Alistipes*, and *Parabacteroides*. Additionally, MetaStat analysis showed that seven bacterial genera, *Bacillus*, *Lactobacillus, Marivita*, *Limosilactobacillus*, and *Psychrobacter,* were enriched in the control group. *Helicobacter* and *Candidatus Bacilloplasma* were aggregated in the CPP-1 group (Figure 5D). LEfSe difference analysis was performed to determine the effect of CPP-1 on the abundance of each species in the differential flora, with a linear discriminant analysis (LDA) threshold of 4.0. The results illustrated that the CPP-1-treated group was enriched in *Candidatus_Bacilloplasma*, *Mycoplasmataceae*, and *Mycoplasmatales*, and the control group was abundant in *Staphylococcales*, *Staphylococcaceae*, *Moraxellaceae*, and *Acinetobacter* (Figure 5E).

### 3.4. CPP-1 Alleviated the Oxidative Stress, Inflammatory Responses, and Lipid Metabolism in Liver of Naturally Aging Mice by Intestinal Microbe-Related Gut–Liver Axis

**Figure 6:**
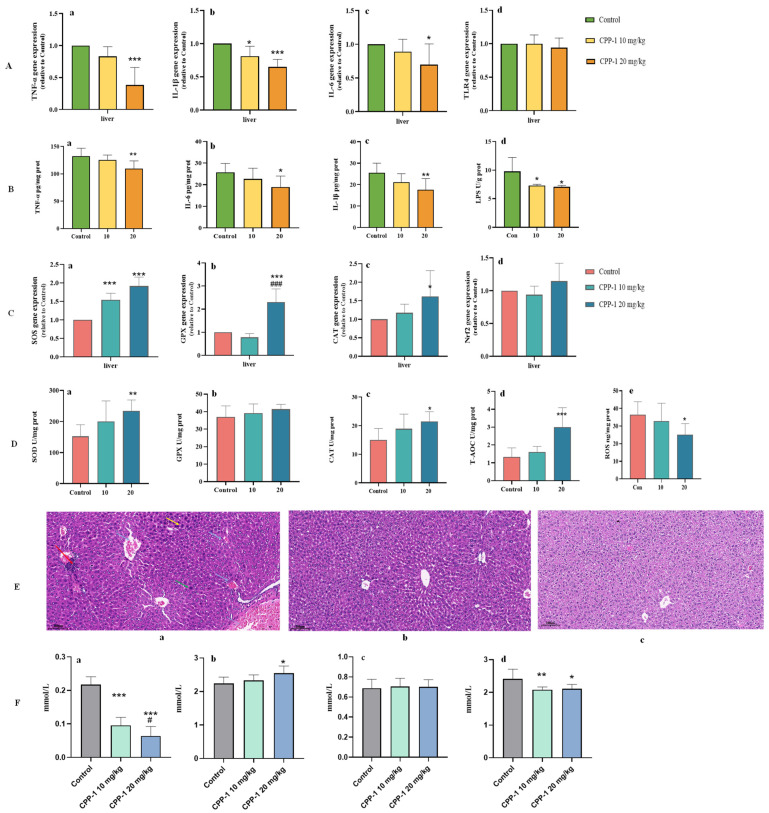
Effects of CPP-1 on anti-oxidant, anti-inflammatory, and liver lipid metabolism dysfunction recovery capacity of liver in naturally aging mice. (**A**) The gene expressions of inflammatory factors, (**a**) TNF-α, (**b**) IL-1β, (**c**) IL-6, and (**d**) TLR4; (**B**) the protein levels of inflammatory factors, (**a**) TNF-α, (**b**) IL-6, (**c**) IL-1β, and (**d**) LPS; (**C**) the gene expressions of anti-oxidative enzymes, (**a**) SOD, (**b**) GPX, (**c**) CAT, and (**d**) Nrf2; (**D**) the protein levels of anti-oxidative enzymes (**a**) SOD, (**b**) GPX, (**c**) CAT, (**d**) T-AOC, and (**e**) ROS; (**E**) the histopathological staining of liver, (**a**) Control, (**b**) CPP-1 10 mg/kg, (**c**) 20 mg/kg. Red arrow: inflammatory cell infiltration; blue arrow: erythrocyte stasis or protein-like material deposition; green arrow: hepatocellular swelling; yellow arrow: binuclear hepatocyte; (**F**) quantification shows the serum levels of (**a**) LDL-C, (**b**) HDL-C, (**c**) TC, (**d**) TG of different groups of mice; error bars indicate SEM (*n* = 6). Differences are statistically significant at * *p* < 0.05, ** *p* < 0.01, *** *p* < 0.001 compared with natural control group. # *p* < 0.05, ### *p* < 0.001 compared with 10 mg/kg CPP-1 group.

When the intestinal barrier is compromised, there is increased translocation of bacterial-derived microbe-associated molecular patterns (MAMPs) to the liver, and MAMPs can bind to pattern-recognition receptors (PRRs, such as TLR4, NOD1/2) on hepatocytes, leading to the activation of pro-inflammatory and pro-oxidative signaling cascades [32]. And according to the results of Section 3.1, CPP-1 could reduce the serum levels of ALT and AST, indicating that CPP-1 has a restorative effect on the liver function of aging mice. Thus, it was inferred that CPP-1 may improve liver function and delay aging by modulating intestinal flora abundance and diversity, as well as the levels and composition of bacterial-derived metabolites. Initially, we determined the relative gene expressions and protein levels of inflammatory cytokines and oxidative enzymes of aging mice to evaluate the anti-inflammatory and anti-oxidative effects on the liver of CPP-1 through the microbe-related gut–liver axis. As shown in Figure 6A,B, CPP-1 significantly and dose-dependently reduced the relative gene expressions and protein level of inflammatory cytokines, including TNF-α, IL-1β, and IL-6, and the gene expression of TLR4. It suggested that the high-dose CPP-1 had a greater anti-inflammatory effect on the liver, which was consistent with the results of the intestine. The LPS level was elevated in the liver of aging mice since the intestinal barrier was damaged, and LPS entered the liver through the blood circulation (gut–liver axis). Regarding the anti-oxidative aspect (Figure 6C,D), the relative gene expressions of SOD, CAT, GPX, and Nrf2 and the protein levels of SOD, CAT, GPX, and T-AOC were enhanced and the level of ROS was decreased by CPP-1 in comparison to the aging mice, indicating CPP-1 could protect the liver against aging by alleviating oxidative stress, and the high-dose CPP-1 group exhibited greater advantages for delaying the aging process in terms of inflammation and oxidative stress.

As shown in the images of the histological morphology of the liver (Figure 6E(a–c)), the aging mice in the natural control group exhibited aging lesions, including enlarged hepatocytes (green arrow), binucleated hepatocytes (yellow arrows), inflammatory cell infiltration (red arrows), and erythrocyte sludge or protein-like material accumulation (blue arrows). In contrast, these symptoms were effectively alleviated in the CPP-1 supplementation group, especially the inflammatory infiltration, suggesting that CPP-1 treatment attenuated the liver injury caused by aging via reducing inflammatory damage.

To further study the anti-aging mechanism of CPP-1 on lipid metabolism in the liver, several indices were determined, including LDL-C, HDL-C, TC, and TG, and the results are shown in Figure 6F. When compared with the effects in the young mice, significant increases in LDL-C, TC, and TG and a substantial decrease in the HDL-C levels were observed in the aging mice, which suggested that the aging mice suffered from dysregulated lipid metabolism [33]. Interestingly, these pathologic changes could be affected by the treatment of CPP-1. In those mice treated with CPP-1, the LDL-C and HDL-C levels were significantly decreased and increased, respectively, compared with the aging mice. Furthermore, CPP-1 down-regulated the serum level of TG significantly. However, the serum level of TC was not decreased obviously by CPP-1. These results indicated that 20 mg/kg CPP-1 was superior to 10 mg/kg CPP-1 in the remission of aging-related lipid metabolism.

### 3.5. Correlation Analysis between Inflammatory Factors and Oxidative Enzymes and Intestinal Microbiota

**Figure 7:**
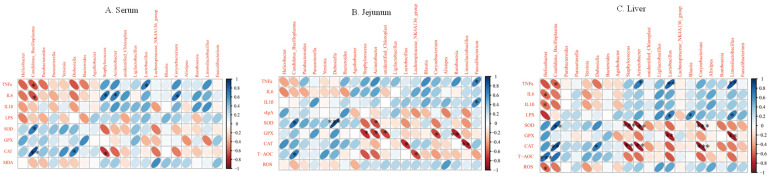
Heatmap showing the correlation of gut microbial genera with pro-inflammatory factors (IL-6, IL-1β, TNF-α), anti-oxidant enzymes (SOD, GPX, CAT), MDA, sIgA, LPS, ROS, T-AOC. (**A**) Indicates correlation with serum; (**B**) indicates correlation with jejunum; (**C**) indicates correlation with liver. The color and shape of ellipse correlate with the strength of the correlation, with darker and flatter ellipses being more strongly correlated. * *p* < 0.05, ** *p* < 0.01, *** *p* < 0.001.

**Figure 8:**
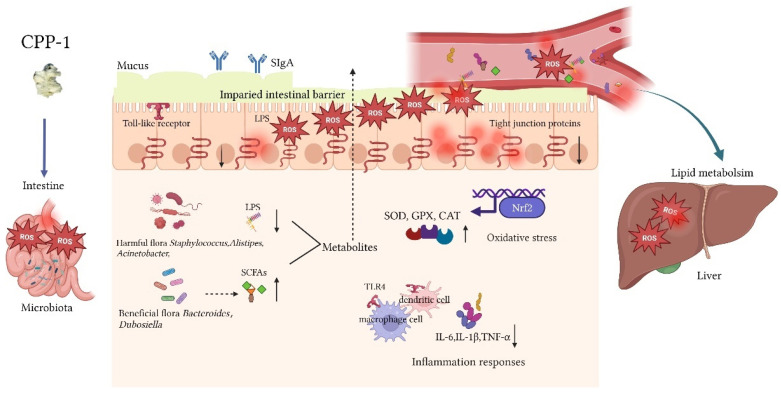
CPP-1 working model in the naturally aging mice. CPP-1 alleviated pathological damage to the gut and liver, including inflammation responses, oxidative stress, intestinal barrier, and lipid metabolism levels by modulating intestinal microbiota-related gut–liver axis. “↑” represents the content was up-regulated by CPP-1, “↓” represents the content was down-regulated by CPP-1.

According to the above results, we observed that alterations in the intestinal metabolite can affect the functions of the intestine and liver. Therefore, we further studied the relationship between intestinal microorganisms and inflammation and oxidative stress via Spearman analysis as a way to elucidate how CPP-1 protects the damaged intestine and liver in naturally aging mice through the intestinal microbe-related gut–liver axis. We performed the correlation analysis using the top twenty abundance of intestinal microorganisms at the genus level with pro-inflammatory cytokines and anti-oxidative enzymes in the serum (Figure 7A, LPS and MDA distinctively), jejunum (Figure 7B, sIgA distinctively), and liver (Figure 7C, LPS distinctively). We observed that the levels of pro-inflammatory factors (TNF-α, IL-6, and IL-1β), LPS, ROS, and MDA had negative correlations with the intestinal microbes *Candidatus_Bacilloplasma* and a positive relationship with *Staphylococcus, Acinetobacter, Lactobacillus*, *Corynebacteriuma,* and *Limosilactobacillus*. Furthermore, the activity of anti-oxidative enzymes (SOD, GPX, and CAT), sIgA, and T-AOC had a negative correlation with *Staphylococcus, Acinetobacter, Corynebacterium, Lactobacillus*, and *Limosilactobacillus* and positive relationships with *Candidatus_Bacilloplasma* and *Dubosiella.* These results revealed the correlations between certain intestinal flora and inflammatory factors and anti-oxidant enzymes, suggesting that the intestinal microbiota plays a critical role during the delaying of the aging process. As shown in Figure 8, during aging, the disordered intestinal flora disrupted the microbial environment of the intestine and metabolites, and with the damage to the intestinal barrier, the microorganisms and their metabolites diffused into the bloodstream, thus affecting the liver via the gut–liver axis. Polysaccharides CPP-1, as macromolecules, were degraded and utilized by intestinal microbes to affect their metabolites and secretion, repairing the intestinal barrier and improving the intestinal and liver function of naturally aging mice via the intestinal flora-associated enterohepatic axis.

## 4. Discussion

Aging is the gradual deterioration of the body’s various physiological functions, which eventually leads to death. The body’s resistance to external stresses gradually declines during this process and diseases associated with aging gradually increase [34]. CPP-1, a natural polysaccharide fraction extracted from *C. pilosula* roots, has been identified as a typic plant pectin in our previous study [22]. Polysaccharides are mostly digested in a mammal’s intestine and degraded by gut microbiota; thus, it is hypothesized that the intestine is the major target organ of polysaccharides, which can protect against intestinal injury by modulating the intestinal microbiota, barrier, and immune responses [35]. A number of studies have shown that the intestinal microbiota, which has been described as an invisible “organ”, has a close and coordinated relationship with the gut and liver [36]. The intestinal mucosal barrier either acts as a physical barrier or coexists with microbiota. Once the symbiotic equilibrium is disrupted, microbiota respond to this imbalance; for instance, some bacteria-derived metabolites (LPS, fatty acids) circuited into the liver to affect the hepatic functions (lipid metabolism, inflammatory responses, and oxidative stress), which is also known as the gut–liver axis [36]. Therefore, the anti-aging effect of CPP-1 on naturally aging mice was studied, focusing on evaluating the intestinal and hepatic functions via the gut microbe-related intestine–liver axis (Figure 8).

Oxidative stress impairs naturally aging and the protection against oxidative stress is a common mechanism mediating the phenotype observed in animal models of longevity [37]. Therefore, medicine that alleviates oxidative damage is the first choice for delaying aging. The existence of anti-oxidant enzymes could reduce the generation of lipid peroxides and hinder oxidation, which plays a critical role in maintaining the balance between the oxidation and anti-oxidation defense system [38]. SOD catalyzes the dismutation (or portioning) of the superoxide radical into ordinary molecular oxygen. CAT is a normal enzyme that catalyzes the decomposition of H_2_O_2_ to water and oxygen; thus, the activity of CAT could reflect the ability to remove ROS and the resistance to oxidative stress. GPX could protect the biofilms from ROS damage and maintain the cell function [39]. MDA is the most important end-product of lipid peroxidation, which reflects the level of lipid peroxidation [40]. As a result, the shifts in anti-oxidant enzymes and MDA levels will break the balance between the oxidation and anti-oxidation defense systems, contributing to the excessive accumulation of ROS, lipid peroxidation of cells, oxidation of membrane phospholipids, and finally, lead to cell apoptosis. Furthermore, Nrf2 is an important transcriptional master regulator that is considered to recognize cellular oxidative stress and combine with the anti-oxidative enzyme to enhance the anti-oxidative reactions that participated in sustaining the redox state of cells [41]. In our study, the results demonstrated that CPP-1 could alleviate the oxidative stresses by increasing the levels of anti-oxidative enzymes and reducing ROS and MDA with dose-dependence in the intestine, liver, and serum, suggesting that CPP-1 could delay aging in naturally aging mice by repairing the oxidative damage in the intestine, serum, and liver.

Age-related oxidative stress leads to the activation of inflammatory system molecules, according to the molecular inflammatory theory of aging [42]. Aging disrupts the equilibrium between pro- and anti-inflammatory cytokines, which favors an excessive production of IL-6, TNF-α, and IL-1β [43]. It is generally accepted that the increased inflammation in older individuals may lead to age-associated disease, making it a disease marker [42]. Therefore, research into anti-inflammatory medications that can delay aging is essential. The levels of inflammatory factors TNF-α and IL-6 are important aging biomarkers, which increase significantly in old organisms [44]. The pro-inflammatory cytokine IL-1β is a key factor in triggering age-related chronic inflammation [45]. During the aging process, various inflammatory factors (LPS, free fatty acids, ROS) could bind to TLR and active NF-κB, and they are important in the immune system and inflammatory responses [46]. In this study, CPP-1 could decrease the mRNA and protein levels of TNF-α, IL-1β, and IL-6 and the mRNA level of TLR4 in the intestine and liver. Additionally, CPP-1 also reduces the protein levels of inflammatory cytokines in the serum, which indicated that CPP-1 could delay aging by attenuating the inflammatory responses.

Growing shreds of evidence have indicated the composition and diversity of the intestinal microbiota community have a close relationship with the aging process [47,48]. The maintenance of the intestine bacteria ecosystem is vital for the protection of key gut functions and the microbe-related gut–liver axis during the aging process. To study the effect of CPP-1 on the intestinal microbiota of aging mice in natural conditions, the intestinal flora composition was analyzed. At the phylum level, the *Firmicutes/Bacteriodota* value (F/B) has been proposed as an indicator of the overall status of the intestine microbiota. According to the previous study, the F/B in aging mice increased significantly compared with young individuals, while CPP-1 supplementation decreased the F/B value, which was consistent with previous studies [31,49]. *Bacteriodota* first converts pyruvate to acetyl-CoA before using phosphor-acetyltransferase and acetate kinase to produce acetic acids [50]. Furthermore, *Bacterodota* can also produce propionic acid via the acrylic acid pathway. Acetic acid and propionic acid can inhibit TNF-α and inflammation and influence lipid metabolism [50]. As for the genus level, the CPP-1 remedy modulated the abundance and composition of the intestinal flora, including increased *Bacteroide* and decreased *Staphylococcus*, *Alistipes*, *Acinetobacter*, *Parasutterella*, and *Parabacteroides* compared with the aging mice. This also explained the main reason why CPP-1 reduced the intestinal microbiota abundance of aging mice was CPP-1 modulating microbiota primarily through decreasing the abundance of harmful flora. *Bacteroides*, stimulated by CPP-1, increased significantly due to its ability to degrade polysaccharides and provide vitamins and nutrients to the host and other intestinal microbial residents [43,51]. Also, *Bacteroides*, as a kind of short-chain fatty-acid (SCFA, could modulate hepatic lipid metabolism)-producing bacteria, was up-regulated via CPP-1 supplementation, indicating that CPP-1 may affect lipid metabolism via the microbiota-related gut–liver axis [52]. By contrast, *Alistipes* is a relatively new genus of microbes isolated from medical clinical samples, which is highly relevant in dysbiosis and inflammatory diseases [53]. Previous studies showed that *Alistipes* was positively correlated with LPS, serum TC, and TG, indicating it was tightly associated with intestinal inflammation and lipid metabolism [54]. *Staphylococcus* in the gut microbiota was considered to be harmful to the intestinal immune system [29]. *Acinetobacter* (LPS-produced bacteria)*, Parabacteroides,* and *Parasutterella* were recognized as intestinal genera connected with chronic inflammation in the gut [55,56,57].

In this study, CPP-1 decreased the abundance of harmful intestinal bacteria as mentioned above, and was shown to alleviate the inflammation, oxidative stress, and lipid metabolism caused by aging. To sum up, CPP-1 treatment slowed the aging speed of mice by modulating the intestinal microbiota. In addition, based on the Spearman analysis, *Acinetobacter* (flora-produced LPS), and *Staphylococcus* had positive correlations with pro-inflammatory cytokines and negative correlations with anti-oxidative enzymes, while *Dubosiella* (flora-produced SCFA) showed an opposite trend compared to those mentioned above, suggesting that these microbiota may protect the intestine and liver by modulating the intestinal flora environment as well as the composition of metabolites that further affect the gut–liver axis.

In addition, the impairment of the gut epithelial barrier in the context of disease in the organism can induce disturbed immune homeostasis in gut and liver tissues (for instance, gradually increasing inflammatory responses), with further implications on disease progression in both the gut and liver [58]. MUC2 is an important secretory protein found in the human gut, which plays a critical role in the protection of the intestinal barrier [59]. ZO-1 and occludin proteins are significant tight junction proteins in the intestine that exert important effects on the maintenance of the intestinal barrier [60]. SIgA is one important defense line in the intestinal mucosal surface to protect the intestinal epithelium from enteric toxins and pathogenic microorganisms [61]. Gut-derived LPS, which is induced by the increased proportions of harmful microorganisms (such as *Alistipes* and *Acinetobacter* mentioned above), plays a critical role in the resulting intestinal inflammatory responses [62]. In general, the presence of LPS in the intestine of healthy animals is considered harmless [63]; however, once LPS from the gut lumen moves into the circulation, it will strongly stimulate inflammatory reactions at a low concentration. Therefore, the existence of LPS is also a critical index of intestinal permeability [64]. According to our results, in the naturally aging mice, CPP-1 could enhance the levels of MUC2, ZO-1, occludin, and sIgA in the intestine and reduce the level of LPS in the liver and serum, suggesting that CPP-1 could repair the intestinal barrier and intestinal permeability for delaying aging.

In addition, LPS, also known as endotoxins, is a structural compound in the outer membrane of Gram-negative bacteria that induces inflammation through the activation of TLR4 [65]. The increased level of LPS in the blood is defined as metabolic endotoxemia, which will interact with blood lipids in various ways, promoting inflammation, causing lipid metabolism dysfunction, and inducing cardiovascular diseases [65]. In comparison, gut-derived SCFAs (such as *Dubosiella* and *Bacteroides*) have been shown to modulate lipid metabolism through energy expenditure and decreased liver triglyceride accumulation [66,67]. The reason that many interactions in the gut–liver axis are directly or indirectly dependent on the microbiota and its metabolites is because the intestinal barrier is destroyed [58]. We therefore determined the level of regulation of lipid metabolism to evaluate the anti-aging capacity of CPP-1 through the intestinal flora-related gut–liver axis. It is well known that LDL-C is related to a high risk of cardiovascular diseases; in contrast, HDL-C at physiological concentrations could take the cholesterol/cholesterol esters for catabolism via the “reverse cholesterol transport” pathway [33]. The main use of elevated TC and TG levels is to confirm atherosclerosis, blood viscosity, and cardiovascular diseases [68]. The results suggested that CPP-1 may improve hepatic lipid metabolism by modulating the enterohepatic axis associated with intestinal SCFA-producing flora, thereby delaying organismal aging.

Although our study confirmed that CPP-1 has a protective effect on naturally aging mice by reducing oxidative stress and inflammation response of the liver and gut by modulating the intestinal microbiota-related gut–liver axis, this study still has some limitations. The relationship between intestinal bacteria, aging, and CPP-1 is complex and dynamic. Whether the increase and decrease in related microbiota are related to CPP-1 has not been directly proved by more experiments. Furthermore, the degradation process of CPP-1 in the gut is still unknown. Therefore, we need to continue research via in vitro and in vivo experiments in the next step.

## 5. Conclusions

In this study, we obtained polysaccharide CPP-1 from the roots of *C. pilosula*, which was identified as typical plant pectin after structure characterization. Subsequently, different doses of CPP-1 were orally administered to naturally aging mice and the protective effects in terms of oxidative stress and inflammation responses in the intestine and liver were reported. In addition to enhancing intestinal functions by lowering the levels of ROS, pro-inflammatory cytokines, increasing levels of anti-oxidative enzymes, mending the mucosal and immune barriers, repairing structural damage to intestinal tissues, and modulating the composition of the intestinal microbiota, the polysaccharide CPP-1 also had similar effects on liver tissues via the microbiota-related gut–liver axis. In particular, the number of Gram-negative bacteria that produce LPS and leak into the bloodstream decreased, alleviating the oxidative and inflammatory stresses and lipid metabolism contributed via an increase in LPS. Meanwhile, the quantity of beneficial bacteria that produce fatty acids increased, resolving the disorder of lipid metabolism caused by chaotic fatty acids. Thus, it is suggested that CPP-1 delayed aging by ameliorating the oxidative stress and inflammatory responses in the gut and liver via the microbiota-related gut–liver axis. In the future, it is necessary to explore the method of polysaccharides directly targeting the intestinal flora, and intuitively observe the relationship between the polysaccharide and intestinal flora in the anti-aging process.

## Figures and Tables

**Table 1:** The primer sequence.

Gene	Primer Sequence 5′ to 3′	PubMed No.	bp
β-actin	F: CATCCGTAAAGACCTCTATGCCAAC R: ATGGAGCCACCGATCCACA	NM_007393.5	171
IL-1β	F: CCTGTGTTTTCCTCCTTGCCT R: AGTGCGGGCTATGACCAATTC	NM_008361.4	158
TNF-α	F: CTCTTCTCATTCCTGCTCGT R: ACCCCGAAGTTCAGTAGACA	NM_012675.3	62
IL-6	F: AAATATGAGACTGGGGATGTC R: TCAGTCCCAAGAAGGCAAC	NM_001314054	90
TLR4	F: CACTTTATTCAGAGCCGTTG R: AGGCGATACAATTCCAC	NM_021297.3	146
CAT	F: ACCAGATACTCCAAGGCAAA R: TAAAATTTCACTGCAAACCCC	NM_009804.2	137
SOD	F: GAACCATCCACTTCGAGCAG R: ATCACACGATCTTCAATGGAC	NM_011434.2	265
GPX	F: TGCTTGCCTCCTAAATGCTG R: CCCAGAATGACCAAGCCAA	NM_001329860.1	81
Nrf2	F: AACCTCCCTGTTGATGACTTC R: CTGTCGTTTTCTCCCTTTTCTC	NM_001399226.1	101
Mucin 2	F: TCATCAACCTTCACTACCCCA R: TTTTGCACACTAACCCAAC	NM_023566.4	247
ZO-1	F: TCGATCAAATCATTACGACCCT R: GCTCTCAAAACTTCTTCGGTCAA	NM_001352638.1	55
Occludin	F: TTGAAAGTCCACCTCCTTACAGA R: CCGGATAAAAAGAGTACGCTGG	NM_001360536.1	129

## Data Availability

Data are available on request from the authors.

## Supplementary Materials

The following supporting information can be downloaded at: https://www.mdpi.com/article/10.3390/antiox12091781/s1, Supplementary Table S1: The determination of anti-oxidative effect of CPP-1 during aging process in mice.
