# Supplementary material for: A Pectic Polysaccharide from Codonopsis pilosula Alleviates Inflammatory Response and Oxidative Stress of Aging Mice via Modulating Intestinal Microbiota-Related Gut–Liver Axis
Source: Antioxidants (Basel). 2023 Sep 19;12(9):1781. doi: 10.3390/antiox12091781 (PMC10525188; doi:10.3390/antiox12091781)
Supplement: Supplementary file 1 [file antioxidants-12-01781-s001.zip › antioxidants-2573642-supplementary.pdf]

Supplementary

A Pectic Polysaccharide from *Codonopsis pilosula* Alleviates Inflammatory Response and Oxidative Stress of Aging Mice via Modulating Intestinal Microbiota-Related Gut–Liver Axis

Yuanfeng Zou [1,*,†], Hong Yan [1,†], Cenyu Li [1], Fang Wen [1], Xiaoping Jize [1], Chaowen Zhang [1], Siqi Liu [1], Yuzhe Zhao [1], Yuping Fu [1], Lixia Li [1], Fan Liu [2], Ji Chen [2], Rui Li [2], Xingfu Chen [2] and Mengliang Tian [2,*]

[1] Natural Medicine Research Center, College of Veterinary Medicine, Sichuan Agricultural University, Chengdu 611130, China
[2] College of Agronomy, Sichuan Agricultural University, Chengdu 611130, China
* Correspondence: yuanfengzou@sicau.edu.cn (Y.Z.); secondat@sicau.edu.cn (M.T.)
† These authors contributed equally to this work.

Table S1. **The determination of anti-oxidative effect of CPP-1 during aging process in mice.**

	Lot number	Principal	Wave length
SOD	A001-3-2	Xanthine and xanthine oxidase reaction system could produce superoxide anion (O_2^-), SOD can converse O_2^- to H_2O_2 and O_2. Meanwhile, WST-1 could react with O_2^- and generate water-soluble yellow filth.	450 nm
T-AOC	A015-2-1	ABTS is oxidized to green $ABTS^+$ by appropriate oxidants in body. The antioxidants could inhibit the production of $ABTS^+$. The T-AOC level of the sample can be determined by measuring the absorbance of $ABTS^+$ at 405 nm or 734 nm.	405 nm
MDA	A003-1-2	MDA could react with TBA and produce red complex.	523 nm
GSH-PX	A005-1-2	GSH-PX could promote the reaction of H_2O_2 with GSH to form H_2O and GSSG, the activity of GSH-PX can be evaluated by the rate of enzymatic reaction. GSH could react with dinitrobenzoic acid and converse to colored product TNBA at 412 nm.	412 nm
CAT	A007-1-1	The progress of CAT catalyzing H_2O_2 could be suspended by ammonium molybdate. The residual H_2O_2 could react with ammonium molybdate and produce yellow substance.	405 nm
